# The First Live Birth in Lithuania After Application of Preimplantation Genetic Testing

**DOI:** 10.15388/Amed.2020.27.2.5

**Published:** 2020-12-21

**Authors:** Živilė Gudlevičienė, Raminta Baušytė, Evelina Dagytė, Danutė Balkelienė, Algirdas Utkus, Diana Ramašauskaitė

**Affiliations:** Vilnius University Hospital Santaros Klinikos, Division of Fertility Technologies and Germ Cell Bank, Vilnius, Lithuania Vilnius University, Faculty of Medicine, Vilnius, Lithuania; Vilnius University Hospital Santaros Klinikos, Clinic of Obstetrics and Gynaecology, Santaros Fertility Center, Vilnius, Lithuania Vilnius University, Life Sciences Center, Vilnius, Lithuania; Vilnius University Hospital Santaros Klinikos, Center for Medical Genetics, Vilnius, Lithuania Vilnius University, Faculty of Medicine, Vilnius, Lithuania; Vilnius University Hospital Santaros Klinikos, Center for Medical Genetics, Vilnius, Lithuania; Vilnius University, Faculty of Medicine, Vilnius, Lithuania; Vilnius University Hospital Santaros Klinikos, Clinic of Obstetrics and Gynaecology, Vilnius, Lithuania Vilnius University, Faculty of Medicine, Vilnius, Lithuania

**Keywords:** Assisted reproductive technologies, Preimplantation genetic testing, Robertsonian translocation, Intracytoplasmic sperm injection, Embryo biopsy

## Abstract

**Summary. Background.:**

Preimplantation genetic testing (PGT) is a genetic testing procedure that is performed before the implantation of embryos for the identification of genetic abnormalities. It is commonly performed when one or both expecting parents have such abnormalities and are at a high risk of passing them to their offspring. The aim of this case report is to describe the first successful IVF/ICSI/PGT procedure in Lithuania.

**Case report.:**

A 27-year-old woman and a 31-year-old man, a married couple, were referred to VUHSK Santaros Fertility Center after trying to conceive for 4 years. In a previous relationship, the woman got pregnant spontaneously and decided to terminate the pregnancy. The husband does not have any children. During the medical examination, the transvaginal ultrasound revealed a low antral follicle count and low anti-Müllerian hormone level for the woman. Semen analysis for the male patient showed severe oligoastenospermia, which confirmed the previous abnormal spermogram results. Chromosome analysis revealed normal karyotype for the woman (46,XX) and Robertsonian translocation for the husband (45,XY,der(13;14)(q10;q10)). After the interdisciplinary medical team counselling, an ICSI with PGT-SR was suggested for the couple. The woman underwent controlled ovarian hyperstimulation with GnRH antagonist protocol for 11 days. Only one embryo with no unbalanced rearrangements was identified and transferred to the woman. On the 14^th^ day post oocyte retrieval, the first serum β-hCG result was received – 39.5 mIU/ml, and the normal gestational sac at 5 weeks and 3 days was confirmed by ultrasound examination.

**Conclusion:**

the first successful pregnancy was achieved in Lithuania and the first IVF/ICSI/PGT-SR newborn in Lithuania was born in 2019 – a vaginal birth of a healthy girl with gestational age of 38 weeks and 4 days and a weight of 2820 g; the Apgar score was 10/10. The IVF/ICSI/PGT procedure was successfully implemented by the multidisciplinary team in VUHSK.

## Abstract (lt)

**Pirmasis sveikas naujagimis Lietuvoje po preimplantacinio genetinio tyrimo pritaikymo:**

**Santrauka. Įvadas.**

Preimplantacinis genetinis tyrimas****(PGT) – tai procedūra, atliekama su *in vitro* sąlygomis kultivuotais embrionais, siekiant nustatyti genetinius pokyčius iki embriono implantacijos į gimdą. Dažniausiai tokia procedūra atliekama, kai tėvams jau nustatyta genetinių pokyčių ir yra didelė šių pokyčių perdavimo naujagimiui rizika. Šio atvejo aprašymo tikslas – paskelbti pirmąją sėkmingą Lietuvoje atliktą IVF/ICSI/PGT procedurą.

**Atvejo aprašymas.**

Sutuoktinių pora – 27 metų moteris ir 31 metų vyras – atvyko į VULSK Santaros vaisingumo centrą dėl negalėjimo pastoti 4 metus. Moteris buvo pastojusi nuo kito partnerio, tačiau savo noru nėštumą nutraukė. Vyras niekada neturėjo vaikų. Medicininio ištyrimo metu moteriai buvo konstatuotas mažas antralinių folikulų skaičius, žemas antimiulerinio hormono lygis. Vyro spermoje nustatytas nepakankamas spermatozoidų skaičius ir judrumas – ryški oligoastenospermija. Genetinio konsultavimo metu moteriai identifikuotas normalus kariotipas (46,XX), vyrui nustatyta Robertsono translokacija (45,XY,der(13;14) (q10;q10)). Multidisciplininio medicininio konsiliumo metu buvo nuspręsta pritaikyti porai pagalbinio apvaisinimo procedūrą su genetiniu embriono ištyrimu – IVF/ICSI/PGT. Moteriai buvo atlikta kontroliuojama kiaušidžių stimuliacija skiriant GnRH antagonistų 11 dienų. IVF/ICSI/PGT metu gautas vienintelis genetiškai sveikas embrionas, jis perkeltas moteriai į gimdą. 14 parą po kiaušidžių punkcijos moteriai buvo atliktas β-hCG tyrimas iš kraujo, gautas rezultatas – 39,5 mIU/ml – konstatuotas kaip teigiamas nėštumo testas. Nėštumas patvirtintas ultragarsiniu tyrimu po 5 savaičių ir 3 dienų.

**Išvada:**

sėkminga IVF/ICSI/PGT procedūra atlikta ir pirmasis sveikas naujagimis po jos Lietuvoje gimė 2019 metais. Naujagimė – sveika mergaitė, gimė natūraliu būdu, 38 savaičių ir 4 dienų gestacinio amžiaus, 2820 g svorio, pagal Apgar skalę įvertinta 10/10. Intensyviai bendradarbiaujant multidisciplininei specialistų komandai nuo 2017 m., IVF/ICSI/PGT procedūra sėkmingai įdiegta VULSK.

**Raktažodžiai. **Pagalbinio apvaisinimo technologijos, preimplantacinis genetinis tyrimas, Robertsono translokacija, intracitoplazminė spermatozoido injekcija, embriono biopsija

## Introduction

Preimplantation genetic testing (PGT) is a genetic testing procedure that is performed before the implantation of early stage (2-5 days after fertilization in the laboratory) embryos to identify genetic rearrangements and select the best one(s) for the embryo transfer [1, 2]. PGT is divided into PGT for aneuploidies (PGT-A), PGT for monogenic/single gene defects (PGT-M), and PGT for chromosomal structural rearrangements (PGT-SR) [3, 4, 5]. PGT-A is performed for couples of advanced maternal age, or histories of recurrent miscarriage and repeated implantation failure. PGT-M is performed for couples with confirmed monogenic hereditary diseases and when monogenic disease has already been detected in one of family’s children. PGT-SR is commonly performed when one or both partners have genetic chromosomal rearrangements and are at a high risk of passing them on to their offspring [6, 7].

In Lithuania, first IVF (in vitro fertilization) babies were born in 1994 after “medical tourism” to United Kingdom. The first private clinics were established and the first IVF procedure was performed in Lithuania in 1998 [8]. For a long time, only private IVF clinics have existed in Lithuania with a limited number of cycles due to the lack of a Law for Assisted Reproduction and governmental reimbursement for IVF to couples. According to the Act of legislation of the Ministry of Health (1999), any invasive procedure and research on embryos were prohibited. The Law for Assisted Reproduction was accepted by the Lithuanian Parliament in 2016; the invasive PGT procedures on embryos of genetically screened couples with high risk of passing severe genetic disorders to their offspring was included. The first University Hospital IVF center in Vilnius, the University Hospital Santaros Klinikos (VUHSK) with multidisciplinary genetic counselling for couples and PGT for embryos at the Center for Medical Genetics (CMG) was also established in 2016. The first PGT in VUHSK was performed in 2017, however, the first successful pregnancy and live birth after PGT was achieved in 2019.

## Case report

***Couple investigation***. A 27-year-old woman and her 31-year-old husband attended VUHSK the Centre of Obstetrics and Gynecology (COG) Santaros Fertility Center (SFC) in September 2018 with a referral by a gynecologist due to the male’s infertility after trying to conceive for 4 years. In a previous relationship, the woman got pregnant spontaneously, but decided to terminate the pregnancy. The husband had no children. Patients were informed about scientific impact of this case, asked to sign a VUHSK confirmed Informed Consent for the publication of results, and they signed it.

During the medical examination of the infertile couple in VUHSK COG SFC, unfavorable results for conception were found. First of all, the transvaginal ultrasound for the woman revealed a low antral follicle count of 7 (3 on the right ovary and 4 on the left ovary) on the third menstrual cycle day, and her anti-Müllerian hormone (AMH) level was 0.78 ng/ml, also. These clinical criteria predicted probable poor ovarian response. Interestingly, the woman did not have any known risk factors for diminished ovarian reserve and was of a young reproductive age. Additionally, she was diagnosed with hypothyroidism due to high thyroid-stimulating hormone (TSH) level – 4.785 mU/l. So, levo-thyroxine treatment was started immediately after the endocrinologist consultation. Other laboratory and instrumental examination results were without pathologies.

For the man, abnormal semen analysis results were observed in a few recent years: oligoasteno-spermia (in January 2016; sperm count – 6x10^6^/ml, progressively motile spermatozoa – 33%), oli-goastenoteratospermia (in February 2016; sperm count – 4x10^6^/ml, progressively motile spermatozoa – 25%), azoospermia (in July 2018). Due to these results he was advised by a urologist to receive hormonal treatment, but it had no effect. After the performing of a sperm test in the VUHSK OGC Division of Fertility Technologies and Germ Cell Bank, the husband was diagnosed with severe oli-goastenospermia: sperm cell count was 1x10^6^/ml, only immotile spermatozoa were found. All sperm analysis were evaluated according to the World Health Organization reference 2010 [9, 10]. 

**Figure 1.**
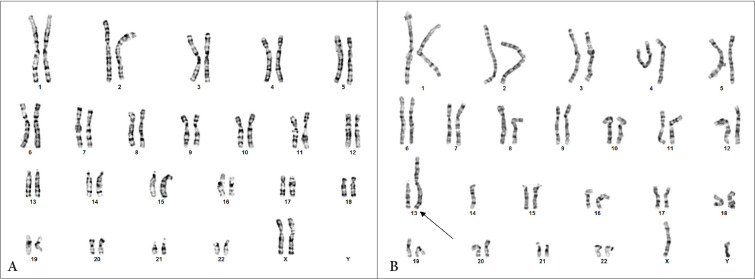
Normal female karyotype (A), male karyotype with Robertsonian translocation (indicated by arrow) (B).

The couple was referred to genetic counselling. Chromosome analysis of G banded metaphases showed normal female karyotype for the woman (46,XX) and male karyotype with balanced chromosomal rearrangement – Robertsonian translocation between 13 and 14 chromosomes for the man (45,XY,der(13;14)(q10;q10)) (Figure 1). 

After interdisciplinary medical team counselling, an IVF procedure with intracytoplasmic sperm injection (ICSI) to be followed by PGT-SR for embryos was suggested. 

***Ovarian stimulation and oocyte retrieval***. IVF stimulation cycle was started for the woman with TSH concentration <2.5 mU/l. After that, on the third menstrual cycle day, she underwent controlled ovarian hyperstimulation with GnRH antagonist stimulation protocol with dose of 300 IU/day of recombinant follicle stimulating hormone (FSH) for 11 days. When the largest follicles reached ≥18 mm, recombinant human chorionic gonadotropin (hCG), a dose of 10000 IU, was provided and 36 h later the transvaginal oocyte retrieval was performed. A total of 13 oocytes were collected, and mature oocytes (in metaphase II) were injected, resulting in viable embryos. After the oocyte retrieval procedure, the luteal phase support with appropriate medications was started. 

**Figure 2.**
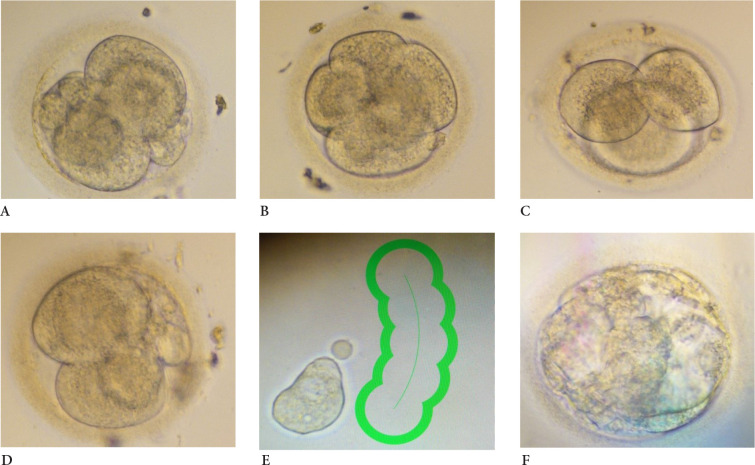
**Embryo development and blastomere biopsy.** **A** – 3 day embryo No 1, 4 cell stage; **B** – 3 day embryo No 2, 6 cell stage; **C** – 3 day embryo No 3, 3 cell stage; **D** 3 day embryo No 4, 4-5 cell stage; **E** – laser assisted single blastomere biopsy; **F** – 5 day poor quality blastocyst from embryo No 2 transferred to the uterus.

***Embryos cultivation***. On the day of IVF under controlled ultrasound ovarian punction using local anaesthesia 13 oocytes were aspirated and directly transferred into FertiCult IVF medium (FertiPro, Belgium). The oocytes were incubated until the processing in 5.5% CO2 and 37°C incubator (Astec, Japan,). 4 hours after aspiration, the oocytes were prepared for ICSI procedure: denuded using 135 mikrom Denuding pipette (Gynetics, Belgium) and 10% Hialuronidase (FertiPro, Belgium). After denudation, only 8 methaphase II (MII) oocytes were injected under an inverted Nikon Eclipse Ti microscope (Nikon, Japan) using RI Integra TM 3 micromanipulator (Research Instruments, UK) and 35° Injection and 35° Holding micropipettes (Reproline, Germany). 6 out of 8 injected oocytes were fertilized after ICSI: the two-pronucleus stage was stated 24 hours after the ovarian punction. Embryos were cultivated in 50 mikrol drops in the one step SAGE medium (Origio, Denmark) under the mineral oil (Irvine Scientific, USA). On day 3 of the embryo development (3-6 blastomere stage, Figure 2 A, B, C, D), the embryo biopsy using RI Saturn laser (Research Instruments, UK), 50 mikrom Biopsy pipettes (Reproline, Germany) and RI Integra TM 3 micromanipulator (Research Instruments, UK) was performed. RI Viewer program (Research Instruments, UK) was used to select and cut the single blastomere (Figure 2 E). The single blastomere was transferred into the 0.2 ml microtubes and directly transported on ice to the VUHSK CMG. 

***Genetic analysis of the embryos***. In the CMG, genetic chromosomal analysis of the embryos was performed by Next Generation Sequencing (on the Ion PGM™ System, Life Technologies, USA) using The Ion ReproSeq™ PGS View Kits (PGT-SR) (Life Technologies, USA). The method is based on whole genome amplification and low-pass whole genome next generation sequencing to detect chromosomal aneuploidies, unbalanced chromosome arm events (>48 Mb) from a single cell.

**Figure 3.**
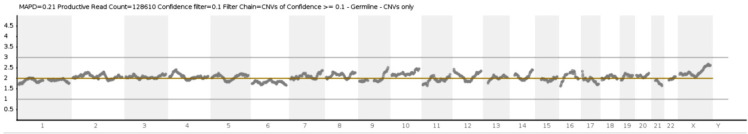
The report visualisation of the PGT-SR analysis of the Next Generation Sequencing on the Ion PGM™ System using The Ion ReproSeq™ PGS View Kits of the 2^nd^ embryo. The embryo was diploid with a 46,XX karyotype.

Four embryos were analysed. Three embryos (1^st^, 3^rd^ and 4^th^) showed a chaotic chromosomal constitution. One embryo (2^nd^) had a normal diploid karyotype with a 46,XX karyotype without any unbalanced chromosome rearrangements >48 Mb (Figure 3). The median absolute pairwise difference (MAPD) value was 0.210. MAPD is an estimate of coverage variability between adjacent genomic tiles. The productive reads count of a low-pass whole genome next generation sequencing was 128 610. The copy number variation (CNV) call at a MAPD <0.300 and the productive reads count >100 000 are classified as accurate. The PGT-SR analysis result of the 2^nd^ embryo was normal. The diploid 2^nd^ embryo was transferred to the uterus.

***Embryo transfer***. After performing PGT-SR, only one embryo with no unbalanced chromosomal rearrangements (embryo No 2, see Figure 2 B) was identified and transferred to the woman on day 5 (Figure 2 F) using Cook Access Nano embryo transfer catheter (Cook, USA).

***Pregnancy and baby birth***. On the 14th day post oocyte retrieval (POD), the first serum β-hCG test result was received at concentration 39.5 mIU/mL. After this result, periodically performed serum β-hCG tests showed a steadily increasing serum β-hCG level: on POD 17 serum β-hCG test result was 154 mIU/mL, on POD 19 – 367 mIU/mL, on POD 21 – 912 mIU/mL. The normal gestational sac at 5 weeks and 3 days was confirmed by ultrasound examination. During the first pregnancy trimester, the woman was followed up in VUHSK OGC SFC with regular ultrasound examinations, treatment correction, and consultations.

Gestational diabetes was diagnosed at 12 weeks of pregnancy; the special meal plan and regular physical activity were prescribed. Every month, the pregnant woman was consulted by a perina-tologist and endocrinologist. The management of gestational diabetes was effective. At 38 weeks of pregnancy due to prelabour rupture of membranes, the women was admitted to the obstetrical department. There was a vaginal birth of a healthy girl with a weight of 2820 g. The Apgar score was 10/10. All screening tests of the new-born were in normal range. On the fourth postpartum day the mother and healthy new-born were discharged from the hospital.

## Discussion

This case report showed that PGT procedures could be successfully implemented by the multidisciplinary team working in close relation and coordination during the all process. First of all, it is worth mentioning, that karyotype and Y-chromosome analysis should be offered to all men with nonobstructive azoospermia or severe oligospermia (<5×10^6^/mL) before performing ICSI with their sperm, because they are at increased risk of having a definable genetic abnormality [*Practice Committee of American Society for Reproductive Medicine in collaboration with Society for Male Reproduction and Urology. Fertil Steril. 2008 Nov; 90(5 Suppl):S121-4.*]. This case report clearly confirmed the recommendation. According to genetic testing, the infertility treatment plan for the couple was significantly adjusted with inclusion of the PGT procedure, which gave an opportunity to successfully conceive after 4 years of attempting to get this. Also, from the gynaecological view, one of the most important step was to choose an appropriately controlled ovarian hyperstimulation protocol and stimulation dose, especially for the prediction of probable poor ovarian response [11, 12] and in the absence of the previous ovarian stimulation for the woman. Some researchers suggest that most young patients reached embryo transfer with one to seven oocytes with a good outcome [13]. On the other hand, it is known that affected embryos and uninterpreted embryos decrease significantly the number of suitable embryos for IVF/ICSI/PGT transfer by 25–75% [13]. Under these circumstances, a GnRH antagonist protocol and higher stimulation dose was chosen.

From the embryologist point of view, it is very important to choose the right day of the embryo biopsy. It is possible to do a biopsy from day 2 (single blastomere) till day 5 (several cells of trophoe-ctoderm biopsy). Different laboratories use different methods for the embryo biopsy. In this case, we performed the biopsy on day 4 of the embryo development in vitro: firstly, due to the slow embryo development, and secondly, in order to have enough time to perform the PGT and transfer the fresh embryo on day 5. Today in the world of assisted reproductive technologies and PGT, more and more microinvasive (aspiration of blastocellic cavity) or noninvasive methods of embryo testing (circulated embryo DNA) are developed and implemented in the quotidian work of ART laboratories [14]. And finally – the experience of the genetic laboratory – another important issue in the process of genetic embryo testing. It is very important to choose the right methods for the couple’s embryo investigation to achieve the best result.

During the woman’s pregnancy after IVF/ICSI/PGT, another new and very important experience was serum β-hCG level monitoring and interpretation. The amount of serum β-hCG in culture media secreted by the blastocysts after biopsy is adversely proportional to the number of trophectoderm cells removed [15, 16]. However, there is a lack of studies on serum β-hCG levels in early pregnancy after the PGD procedure, especially after a blastomere biopsy. We found only one study comparing the mean serum β-hCG concentration between PGT patients (n=129) and age-matched patients who underwent ICSI (n=1161) [17].**The mean serum β-hCG concentration of the PGT group was significantly lower than that of the control group on POD 12, 14 and 21 (37.9 mIU/mL vs 46.9 mIU/ mL, 111.3 mIU/mL vs 145.4 mIU/mL, 2225.0 mIU/mL vs 3614.0 mIU/mL) with the lower cut-off-value of serum β-hCG for predicting a single viable pregnancy of the PGT group on POD 12 and on POD 14 (32.5 mIU/mL and 77.0 mIU/mL) comparing with the control group. The doubling time of serum β-hCG at each time interval showed no significant difference between groups. According to these results, researchers suggested that blastomere biopsy may decrease the β-hCG producing activity of the trophoblasts. In our case, the report of the initial serum β-hCG level, its mean value and doubling time, confirmed this data and supported the demand to set a lower cut-off value of serum β-hCG for predicting pregnancy outcomes and further adequate evaluation in the PGT group.

## Take-home message

The PGT is the only procedure which allows for the identification of genetic abnormalities and for selecting the embryos with balanced karyotype to transfer. In many countries, PGT has become an established reproductive option for people at high risk of having a child affected with a genetic disorder or handicap [18]. PGT is still somewhat controversial in a moral level, indications, and the policy of transferring an affected embryo or uninterpreted embryo, but this method provides a fairly good opportunity for a couple with genetic risk factors to succesfully conceive and carry a pregnancy while avoiding the difficult decision of abortion.

## Conclusion

The IVF/ICSI/PGT procedure was successfully implemented at VUHSK and the first baby undergoing this procedure in Lithuania was born in 2019: a vaginal birth of a healthy girl with a gestational age of 38 weeks and 4 days and a weight of 2820 g was the result; the Apgar score was 10/10.
